# Synovial sarcoma of the brachial plexus – a rare tumor in a rare area: a case report

**DOI:** 10.1186/s13256-018-1860-3

**Published:** 2018-11-09

**Authors:** Sreekanth Raveendran, Albert Abhinay Kota, Edwin Stephen, Samuel C. R. Pallapati, Binu Prathap Thomas

**Affiliations:** 1 Dr. Paul Brand Centre for Hand and Peripheral Nerve Surgery, Vellore, India; 2 Department of Vascular Surgery, Christian Medical College, Vellore, 632004 India

**Keywords:** Synovial cell sarcoma, Soft tissue sarcoma, Brachial plexus

## Abstract

**Background:**

Synovial cell sarcomas are usually seen in a juxta-articular location. However, they occur rarely in the head and neck region.

**Case presentation:**

We report a rare case of brachial plexus synovial sarcoma in a 24-year old South Asian man treated successfully with surgical excision followed by radiotherapy.

**Conclusions:**

Synovial sarcoma arising from the brachial plexus is rare. The treatment is multimodal with complete excision (often challenging owing to the proximity of the neurovascular structures) and adjuvant therapy.

## Background

Synovial sarcomas are malignant soft tissue tumors arising from mesenchymal cells, which resemble synovial cells [1]. They are likely to arise from undifferentiated mesenchymal stem cells and are seen near the large joints of the extremities [2, 3]. Very rarely, they are seen in areas remote from joints and synovial sheaths [4]. Head and neck areas are the most common regions.

Synovial sarcoma of the brachial plexus is extremely rare [5].

Fourteen cases of synovial sarcoma associated with a peripheral nerve have been reported [5]; only six were from the brachial plexus (Table 1) [5–10].

**Table 1:** Synovial sarcoma involving brachial plexus

Case	Age	Sex	Nerves	Size	Treatment	Follow-up	Reference
1	44	F	C5–6 NR	2 cm	Surgery f/b RT and chemotherapy	1 year	Tacconi *et al*. [6] (1996)
2	11	F	C7 NR	0.5 cm	Surgery f/b RT	3 years	Chu *et al*. [7] (2004)
3	11	F	C7 NR	–	Surgery f/b chemotherapy and RT	6 years Recurrence (mortality)	de Ribaupierre *et al*. [8] (2007)
4	10	M	C8 NR	5.5 cm	Surgery f/b RT	6 months	Ghiya *et al*. [5] (2011)
5	53	F	Upper trunk	4.5 cm	Surgery f/b RT and chemotherapy	6 years	Pirouzmand *et al*. [9] (2012)
6	18	F	C7 NR	5.9 cm	Surgery	–	Soomro *et al*. [10] (2016)
7	24	M	C8 NR	13.6 cm	Surgery f/b RT	6 months	Present case

*F* female, *f/b* followed by, *M* male, *NR* nerve root, *RT* radiation therapy

We report a case of synovial sarcoma of the brachial plexus abutting the subclavian artery. The tumor was successfully excised with no functional deficit or recurrence at 6 months. Information about this type of tumor in current medical literature is very limited and hence our work-up plan and treatment plan had to be formulated based on worldwide experience.

## Case presentation

**Fig. 1:**
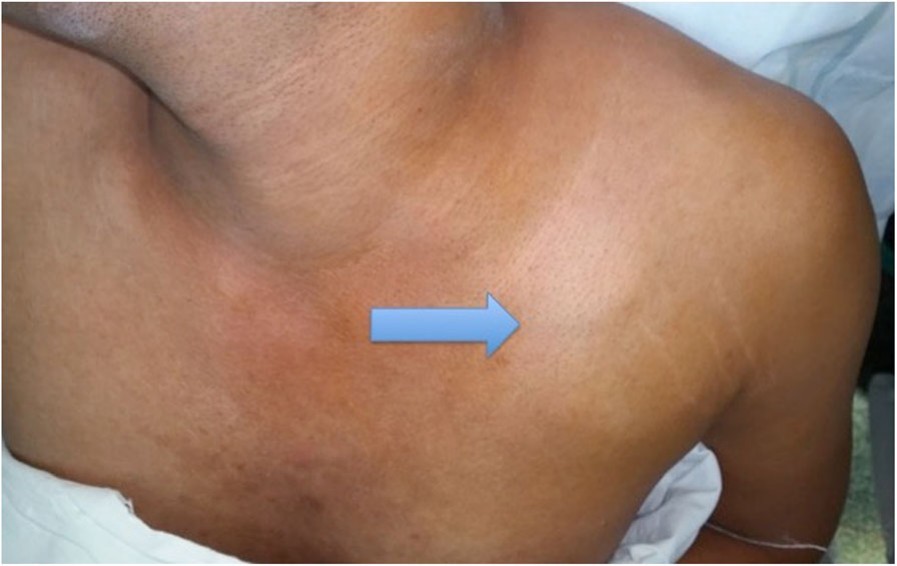
Clinical photograph showing the tumour causing a swelling (arrow) in the infraclavicular region

A 24-year-old South Asian man presented to our hospital with a progressively enlarging swelling that started on the left side of his neck and extended inferior to the clavicle (Fig. 1) increasing in size over a period of 6 months. His opposite upper limb and neck region were normal. He had no co-morbidities.

On examination a 10 × 12 cm globular, firm, non-pulsatile and immobile swelling was palpable on the left side of his neck. Tinel’s sign was negative on percussion. The lateral border of swelling was felt in the apex of axilla; it had smooth lobulated borders. He did not have any motor or sensory deficits. However, the brachial, radial, and ulnar artery pulses were absent. There was no locoregional lymphadenopathy and no metastasis. The clinical staging was stage 3 tumor (T3, N0, M0) according to the tumour, nodes and metastasis (TNM) classification.

**Fig. 2:**
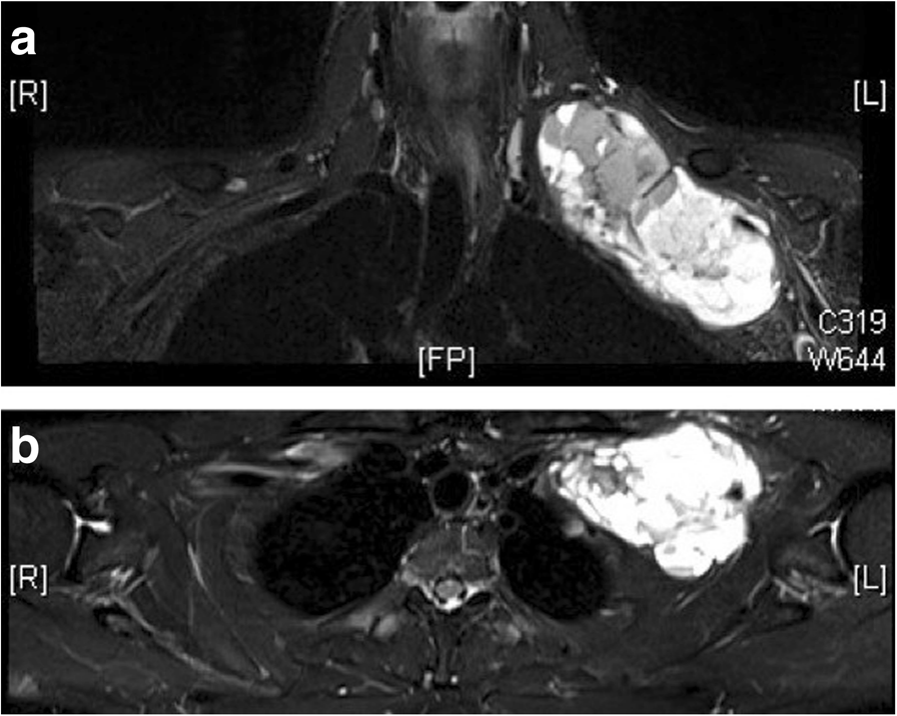
**a** Magnetic resonance T2 coronal image showing hyperintense tumor with hypointense regions in the supraclavicular and infraclavicular region. The subclavian artery can be seen entering the tumor and trunks and cords of brachial plexus can be seen in the cranial and lateral aspect of the lesion. **b** Magnetic resonance T2 transverse image showing tumor deforming first rib and lying close to apex of lung

Magnetic resonance imaging (MRI) showed a well-encapsulated 7.4 cm × 9.2 cm × 13.6 cm, ovoid-shaped, heterogeneous lesion in the left interscalene and posterior triangle, the costoclavicular space, and retropectoralis minor space with hypointense areas on T2/short T1 inversion recovery (STIR) and hyperintense with isointense areas on T1 with fluid levels (Fig. 2). Arterial duplex showed monophasic flow in his distal subclavian artery and vein. An ultrasound-guided biopsy proved the swelling to be synovial sarcoma with positive TLE1, epithelial membrane antigen (EMA), CD56 and CD57 with weak positive S100 and *SYT-SSX1* translocation in immunohistopathology. At a multidisciplinary team (MDT) meeting with medical oncology it was suggested that excision of the lesion be attempted (in view of size and possibility of partial debulking surgery only) followed by adjuvant chemotherapy and radiotherapy (RT).

### Procedure

**Fig. 3:**
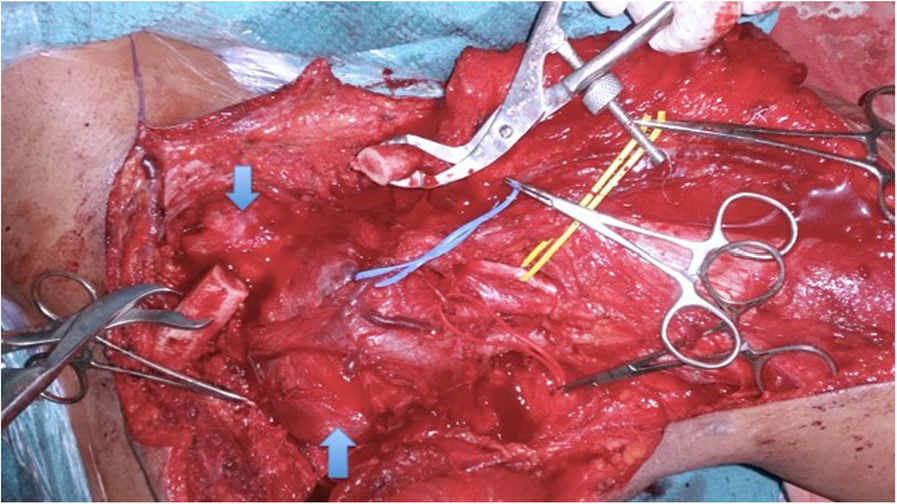
Intraoperative photograph showing the dissected tumor (*arrows*) from the subclavian vein, trunks and cords of the brachial plexus (vessel loops). The tumor was exposed after clavicle osteotomy

The mass was approached through a supraclavicular and infraclavicular approach to the brachial plexus with clavicle osteotomy. Immediately after the clavicle osteotomy the radial pulse was palpable. There was a good dissection plane between tumor and parts of the brachial plexus. The trunks, divisions, and cords of the brachial plexus were in contact with the superior and posterior borders of the mass. The mass arose from the C8 root and lower trunk; the tumor was successfully dissected out from the C8 root and lower trunk. The mass enveloped the mid and distal subclavian artery along its superior-posterior border (Fig. 3). The mass was excised as a whole from the subclavian artery leaving no lesion behind, macroscopically. After the excision of the mass, the clavicle was fixed with a 6-hole dynamic compression plate (DCP). His postoperative period was uneventful. The postoperative volume of the brachial, radial, and ulnar pulses was better than intraoperative volume.

The histopathology was reported as intermediate grade synovial sarcoma with *SYT-SSX1* translocation in immunohistopathology. Since it was near marginal excision and the lesion was of intermediate grade, the oncology MDT meeting decided on adjuvant RT based on the National Comprehensive Cancer Network (NCCN) guidelines [11]. He underwent a full course of RT that included cobalt-60 gamma rays with dose delivered at 66 Gy to mid-plane in 33 fractions. Field size reduction was done after 46 Gy. At 6-month follow-up there were no clinical or radiological signs of recurrence.

## Discussion

Synovial sarcoma accounts for 8% of soft tissue sarcomas [1, 4]. They are high-grade sarcomas and are treated with RT and adjuvant chemotherapy [12]. Three histological types are described, monophasic (fibrous or epithelial), biphasic, and the poorly differentiated (round) type [12]. They are typically seen in young adults [13] and have a better prognosis if they are small enough for marginal excision, with no metastasis, in the extremities, and with *SYT-SSX1* rather than with *SYT-SSX2* [3, 14].

Pluripotent mesenchymal stem cells give rise to synovial sarcomas rather than the synovial membrane, but the name, synovial sarcoma, is due to its resemblance. They have both spindle and epithelioid cells in the biphasic form but only one of them in the monophasic variant. The biphasic variant is easy to detect but the monophasic form has some close differentials like spindle cell carcinoma, hemangiopericytoma, leiomyosarcoma, fibrosarcoma, and melanoma. The use of tumor markers like keratin, vimentin, and EMA, and electron microscopy can confirm the diagnosis [9]. Chromosomal translocations between X and 18, t(X;18)(p11.2; q11.2) are also diagnostic [8, 9].

The goals have been to remove the tumor completely, to control the local growth of the tumor and prevent local recurrence/systemic metastasis. The best management protocol as per current literature for these rare tumors is surgical excision followed by RT. Excision with positive margins is a predictor of local recurrence [11]. Preoperative RT is associated with acute wound complications, hence postoperative RT is usually preferred [11]. NCCN guidelines recommend surgery followed by radiation therapy for stage 3 soft tissue sarcomas that are resectable with acceptable functional outcomes (Level 1 recommendation). The role of adjuvant chemotherapy is still debated and mostly reserved for high grade/unresectable tumors (2B recommendation) [11–13, 15, 16].

Bergh *et al.* reported survival rates at 5, 10, and 15 years of 60%, 50%, and 45% [12].

Our patient was a young man who benefitted from total surgical excision and postoperative RT. At a 6-month follow-up, there was no evidence of recurrence clinically and on MRI.

## Conclusions

Synovial sarcoma arising from the brachial plexus is rare. The treatment is multimodal with complete excision (often challenging owing to the proximity of the neurovascular structures) and adjuvant therapy.
